# Supplementary figures and images for: Hydrogen sulphide alleviates Fusarium Head Blight in wheat seedlings
Source: PeerJ. 2022 Mar 7;10:e13078. doi: 10.7717/peerj.13078 (PMC8908893; doi:10.7717/peerj.13078)

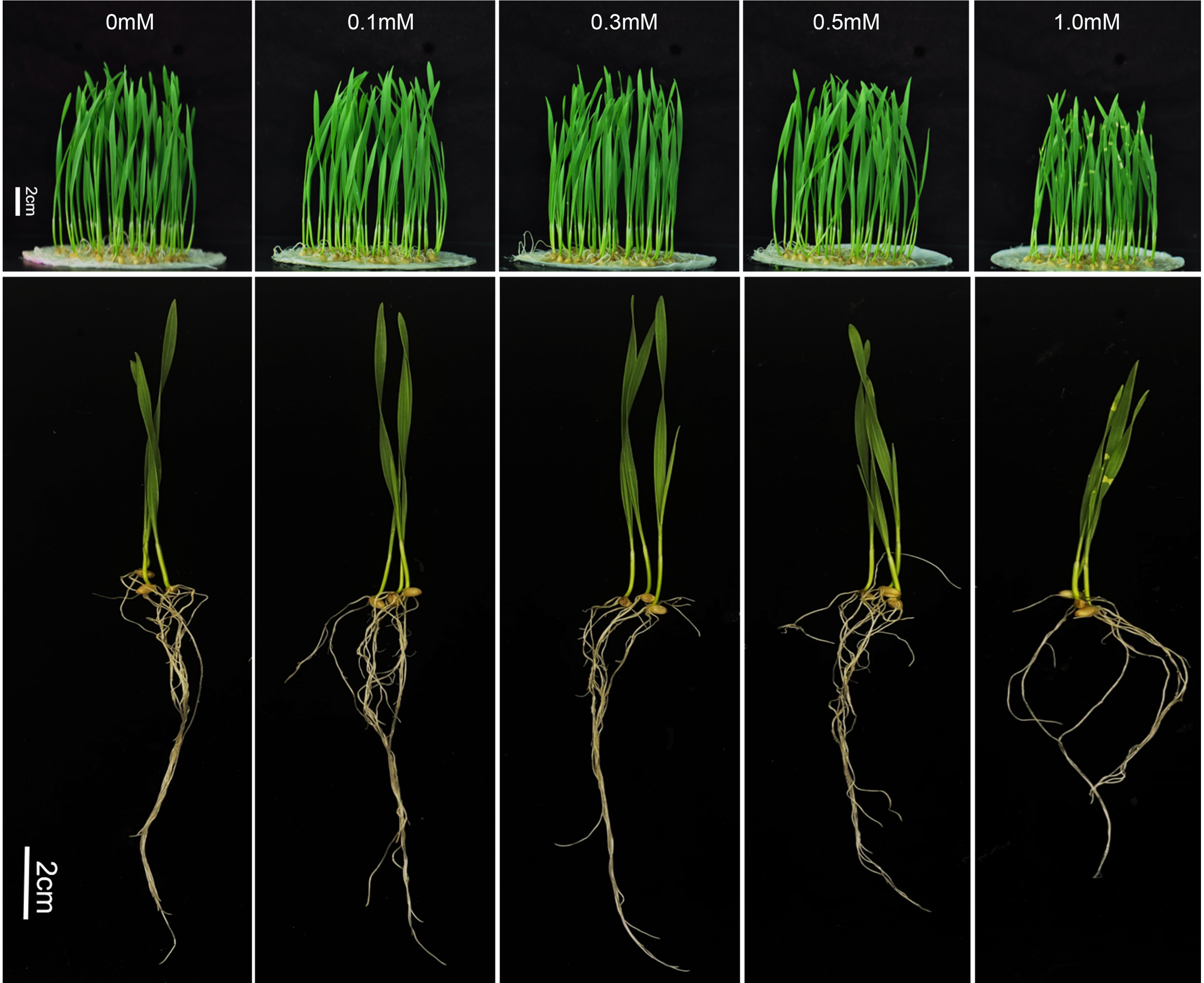

Supplement: Supplemental Information 1 [file peerj-10-13078-s001.jpg]

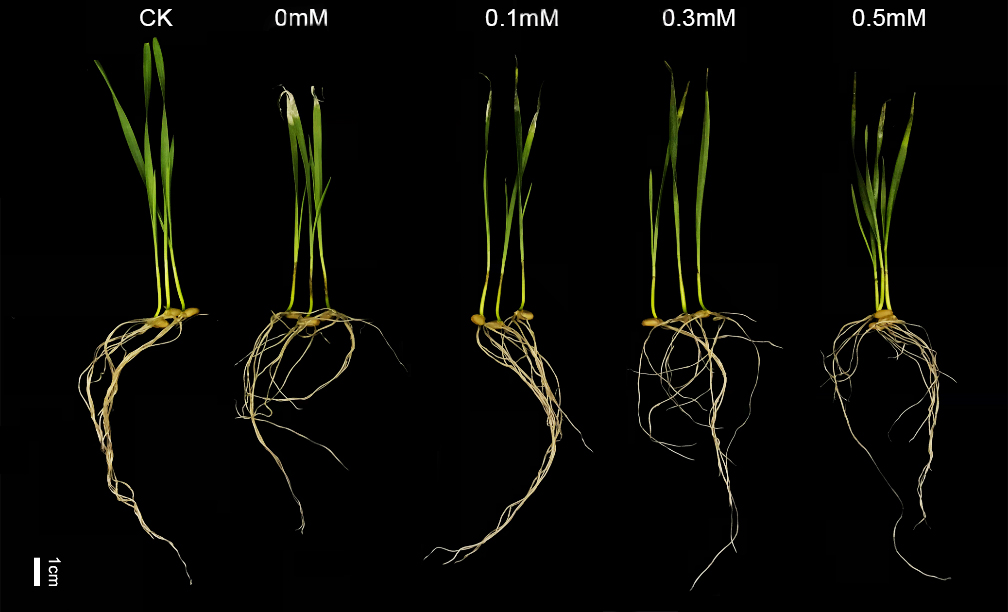

Supplement: Supplemental Information 2 [file peerj-10-13078-s002.jpg]

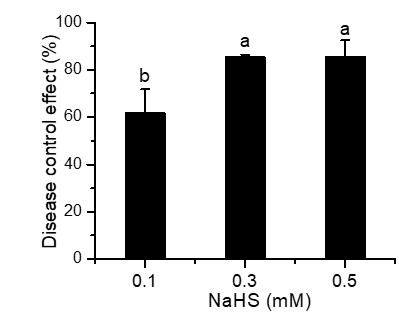

Supplement: Supplemental Information 3 [file peerj-10-13078-s003.jpg]

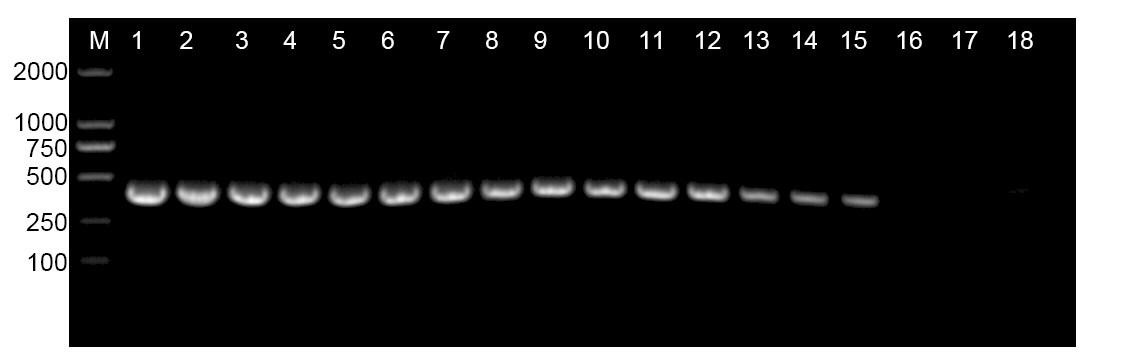

Supplement: Supplemental Information 4 [file peerj-10-13078-s004.jpg]
