# Supplementary material for: Hydrogen sulphide alleviates Fusarium Head Blight in wheat seedlings
Source: PeerJ. 2022 Mar 7;10:e13078. doi: 10.7717/peerj.13078 (PMC8908893; doi:10.7717/peerj.13078)
Supplement: Supplemental Information 10 [file peerj-10-13078-s010.docx]

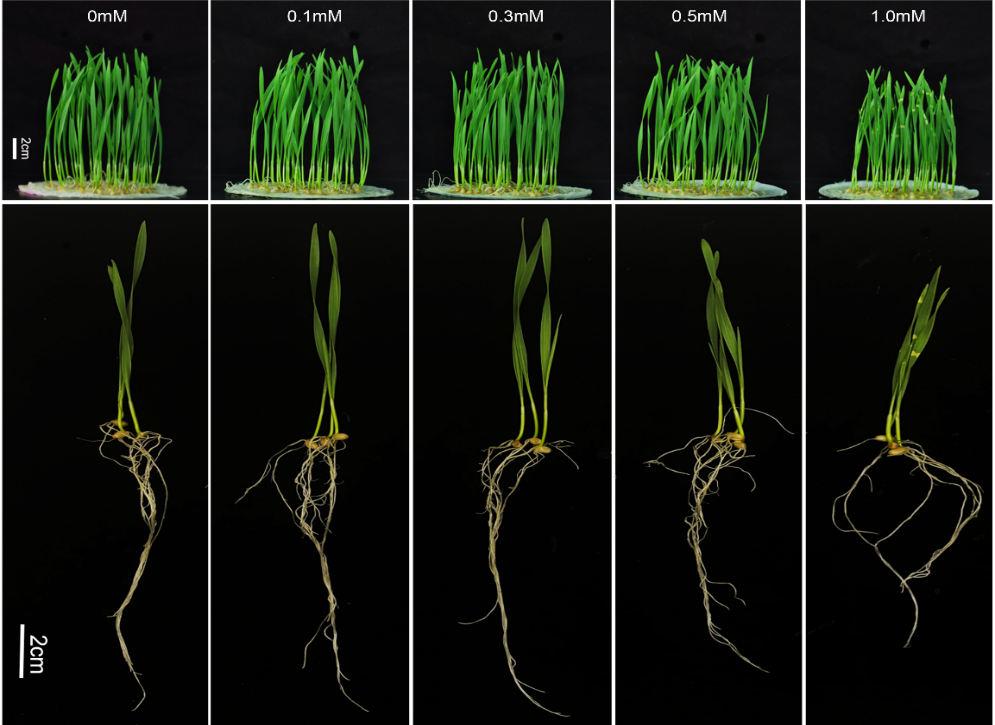


Figure S1. Effects of different concentrations of NaHS treatment on wheat seedlings under normal condition.


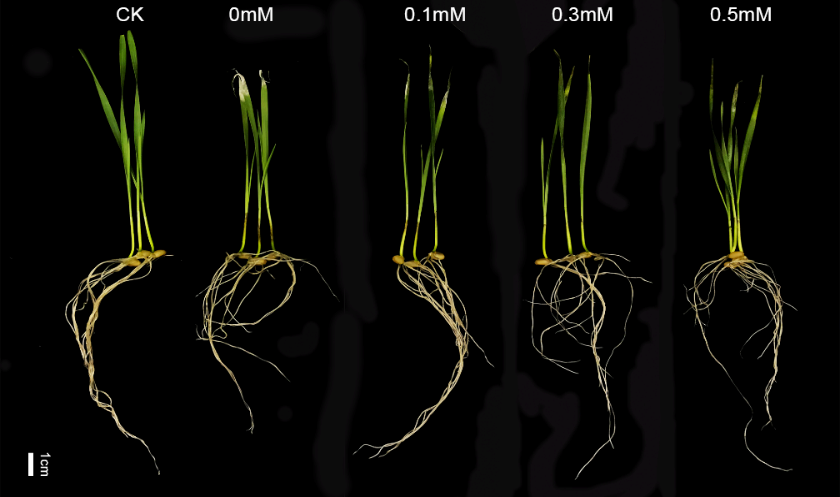


Figure S2. Macroscopic infection phenotypes of representative seedlings treated with different concentrations of NaHS.


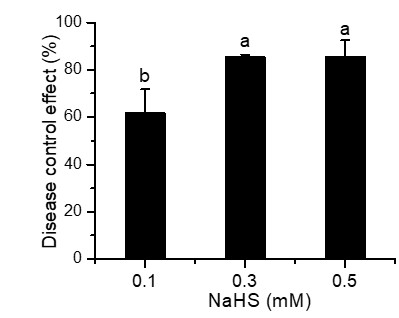


Figure S3. Controlling effect of different concentrations of NaHS against *F. graminearum*.


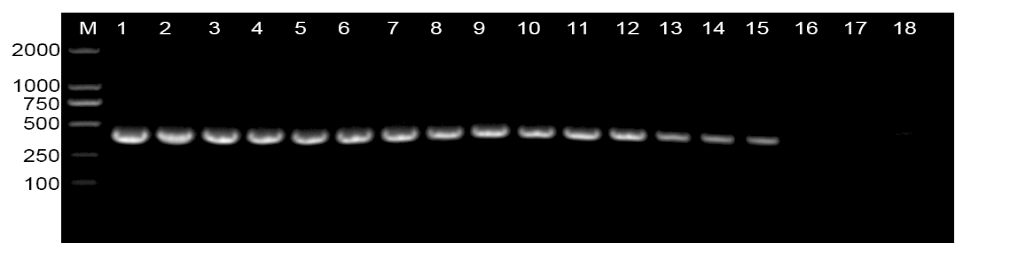


Figure S4. 1.5% of agarose gel electrophoresis of PCR products of fungal DNA from wheat seedlings treated with different concentrations of NaHS with Fg16F/R primer. M, marker; Lanes 1- 3, frozen mycelium from PDA; Lanes 4-6, 0 mM; Lanes 7-9, 0.1 mM; Lanes 10-12, 0.3 mM; Lanes 13-15, 0.5 mM; Lanes 16-18, CK: healthy control.

Table S1. Primer used in materials and methods 2.10.

| Gene symbol | GeneBank accession No. | Anonation | Forward primer sequence (5’-3’) | Reverse primer sequences (5’-3’) | Product size (bp) |
| --- | --- | --- | --- | --- | --- |
| *PR-1.1* | AJ007348.1 | pathogenisis-related protein 1.1 | CTTTCCCAAGCCCAGAACTCG | CGTCCACCCACAGCTTCACC | 249 |
| *PR2* | Y18212.1 | glucan endo-1,3-beta-D-glucosidase | CTCGACATCGGTAACGACCAG | GCGGCGATGTACTTGATGTTC | 119 |
| *PR3* | AB029934 | chitinase 1 | AGAGATAAGCAAGGCCACGTC | GGTTGCTCACCAGGTCCTTC | 116 |
| *PR4* | AF092123.1 | pathogenesis-related protein 4 | TGTCGTGGCGGTCCAAGTA | GTGAAGACGGTGTCCCAGTC | 128 |
| *TaGAPDH* | 7579063 | The glyceraldehyde-3-phosphate dehydrogenase  gene from T. aestivum | CCACTAACTGCCTTGCTCCTCTTG | CTTCCACCTCTCCAGTCCTTGCT | 142 |
| *Fg16* | Specific primers of *F. graminearum* | | CTCCGGATATGTTGCGTCAA | GGTAGGTATCCGACATGGCAA | 400-500 |
